# Type-1 Cannabinoid Receptors Reduce Membrane Fluidity of Capacitated Boar Sperm by Impairing Their Activation by Bicarbonate

**DOI:** 10.1371/journal.pone.0023038

**Published:** 2011-08-04

**Authors:** Barbara Barboni, Nicola Bernabò, Paola Palestini, Laura Botto, Maria Gabriella Pistilli, Marco Charini, Enzo Tettamanti, Natalia Battista, Mauro Maccarrone, Mauro Mattioli

**Affiliations:** 1 Department of Biomedical Comparative Sciences, University of Teramo, Teramo, Italy; 2 Department of Experimental Medicine, University of Milano-Bicocca, Milano, Italy; 3 Department of Food Science, University of Teramo, Teramo, Italy; 4 European Center for Brain Research (CERC)/Santa Lucia Foundation, Rome, Italy; State Key Laboratory of Reproductive Biology, Institute of Zoology, Chinese Academy of Sciences, China

## Abstract

**Background:**

Mammalian spermatozoa acquire their full fertilizing ability (so called capacitation) within the female genital tract, where they are progressively exposed to inverse gradients of inhibiting and stimulating molecules.

**Methodology/Principal Findings:**

In the present research, the effect on this process of anandamide, an endocannabinoid that can either activate or inhibit cannabinoid receptors depending on its concentration, and bicarbonate, an oviductal activatory molecule, was assessed, in order to study the role exerted by the type 1 cannabinoid receptor (CB1R) in the process of lipid membrane remodeling crucial to complete capacitation. To this aim, boar sperm were incubated *in vitro* under capacitating conditions (stimulated by bicarbonate) in the presence or in the absence of methanandamide (Met-AEA), a non-hydrolysable analogue of anandamide. The CB1R involvement was studied by using the specific inhibitor (SR141716) or mimicking its activation by adding a permeable cAMP analogue (8Br-cAMP). By an immunocytochemistry approach it was shown that the Met-AEA inhibits the bicarbonate-dependent translocation of CB1R from the post-equatorial to equatorial region of sperm head. In addition it was found that Met-AEA is able to prevent the bicarbonate-induced increase in membrane disorder and the cholesterol extraction, both preliminary to capacitation, acting through a CB1R-cAMP mediated pathway, as indicated by MC540 and filipin staining, EPR spectroscopy and biochemical analysis on whole membranes (CB1R activity) and on membrane enriched fraction (C/P content and anisotropy).

**Conclusions/Significance:**

Altogether, these data demonstrate that the endocannabinoid system strongly inhibits the process of sperm capacitation, acting as membrane stabilizing agent, thus increasing the basic knowledge on capacitation-related signaling and potentially opening new perspectives in diagnostics and therapeutics of male infertility.

## Introduction

The spermatozoa of eutherian organisms reside within the female genital tract for long time, from hours to days depending on the species, in order to reach a fully fertilizing ability [1]. During this period, the extracellular milieu changes, and progressively the seminal fluid is replaced by the uterine and tubal fluid, so that the sperm cells interact with the female genital tract [2], [3]. Male gametes exposure to these different environments activates a series of biochemical events, collectively termed capacitation, leading to fertile spermatozoa. The intracellular calcium concentration rises [4], [5], the protein phosphorylation pattern changes [1], [6], the actin cytoskeleton reorganizes [7], [8], the motility is hyperactivated [9], the plasma membrane (PM) and the outer acrosome membrane (OAM) become less stable thus acquiring the ability to fuse with each other [10], [11]. In particular, the latter event is mandatory for the completion of the acrosome reaction (AR), that is triggered only after the remodelling of plasma membranes that leads to increased membrane fluidity and to reorganization of specific microdomains where the transduction signalling machinery is segregated [12]–[14]. In this context, the high levels of bicarbonate present in the oviduct or in *in vitro* capacitation media [15] play a key role leading to sperm adenylyl cyclase activation [16]. Then, the cAMP-pathway is involved in the activation of a phospholipid scramblase, responsible for the plasma membrane phospholipids redistribution [17], [18], and for the decrease in phospholipids/cholesterol ratio [11]. This reorganization of membrane phospholipids appears to precede cholesterol efflux driven by soluble protein acceptors, as well as lipid raft migration over the anterior sperm head [19]–[21]. Once all these membrane events are correctly integrated and spatio-temporally regulated, thanks to the dialogue between activating and inhibiting agents, the downstream signalling pathways leading to AR become operational [12]–[14]. Recently, a new set of molecules, belonging to the endocannabonid system, have been identified as modulators of capacitation. Boar and human spermatozoa, in fact, contain anandamide (AEA), a major endocannabinoid that can either activate or inhibit type-1 cannabinoid receptors (CB1R) depending on its concentration [XX]. Additionally, they express a complete and functional biochemical machinery required to synthesize (AEA-synthesizing phospholipase D; NAPE-PLD), degrade (fatty acid amide hydrolase; FAAH), and transport (purported AEA membrane transporter; AMT) AEA, along with the binding receptors CB1R and transient receptor potential vanilloid-1 (TRPV1) [22]–[24]. When high extracellular levels of this bioactive lipid or its non-hydrolysable analogue methanandamide (Met-AEA) are maintained during *in vitro* capacitation of spermatozoa from different species, the acquisition of the fertilizing ability is strongly inhibited [25]–[27]. Combining these experimental results with the evidence of decreasing AEA levels within the female genital tract [28], [29], and the infertility consequence of chronic use of esocannabinoid derivates [30]–[32], the hypothesis of a physiological and pathological role of AEA and analogues in sperm male activation has been proposed; yet, the underlying intracellular pathways remain to be clarified. Looking at other cellular models, the negative influence of AEA on sperm capacitation may be interpreted at least through two different mechanisms: the maintenance of low intracellular cAMP levels [22] through the activation of CB1R, a G_i/o_ associated receptor [33]; or the direct interference of AEA with the dynamic process of membrane microdomains assembly [34], that is so important for the success of sperm capacitation.

Starting from these premises, the present research was designed first to study whether CB1R membrane distribution and activity change during the process of *in vitro* capacitation, elicited in the presence or absence of Met-AEA. Then, a CB1R-mediated involvement of AEA in the process of lipid membrane re-organization, a crucial event in sperm capacitation, was evaluated. To this aim, spermatozoa were cultured under conditions able to up or down regulate phospholipids disorder (with or without bicarbonate, bic) and sperm cholesterol efflux (with or without cholesterol acceptors such as BSA or methyl-β-cyclodextrin, MCD) in the presence of Met-AEA or of the specific CB1R antagonist SR141716 (SR1) [22]. The effects of the different treatments on the process of lipid remodelling were, then, evaluated by analyzing specific physic-chemical membrane parameters.

## Results

### Immunocytochemical localization of CB1R

**Figure 1:**
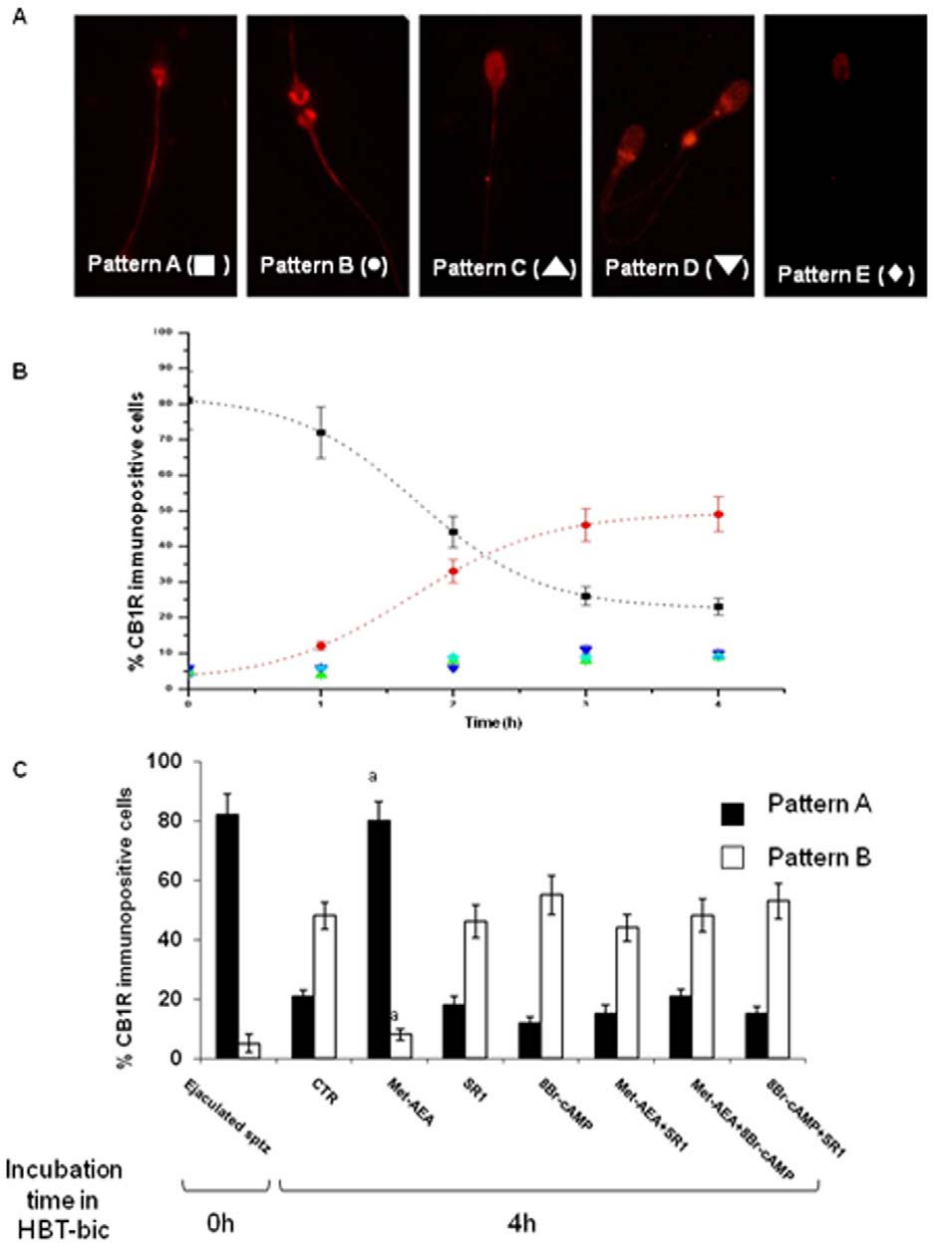
CB1R immunoreactive patterns and their changes during the incubation under different treatments. Panel A) Different patterns of CB1R immunoreactivity found in boar spermatozoa (see text for details). Panel B) Percentage of spermatozoa showing the different CB1R immunoreactivity patterns at different incubation times. Panel C) Effect of different treatments on the percentage of CB1R immunoreactivity patterns A and B, at the beginning (Ejaculated sptz) and at the end of culture (In vitro incubated sptz). ^a^ = p<0.01 vs. other 4 h samples and p>0.05 vs. Ejaculated sptz, ANOVA test.

Immunocytochemical analysis showed that CB1R distribution over sperm membrane changed during the incubation and in relation to the adopted culture conditions (Fig. 1A).

In fact, while more than 80% of ejaculated and incubated spermatozoa in HBT (Hepes buffered Tyrode's solution) displayed CB1R in the post-acrosomial region (Pattern A: Fig. 1A), only 20% of HBT-bic sperm cells maintained this post-acrosomal CB1R localization.

In HBT-bic medium, half of sperm cells re-localized the cannabinoid receptor over the equatorial region (Pattern B: Fig. 1A) at the end of incubation. The incidence of pattern A and B revealed a sigmoidal and inverse relation (R^2^ = 0.996, p<0.001 and 0.998, p<0.001 respectively), when recorded over 1 h interval. When HBT-bic incubated cells were exposed to the physiological inducer of the acrosome reaction, sZP, the incidence of pattern B was reduced by ∼20% (p<0.01 vs. HBT-bic at 4 h: data not shown).

However, the CB1R re-distribution over the equatorial region was completely prevented in Met-AEA incubated spermatozoa. In Met-AEA treated spermatozoa, only the addition of SR1 or 8Br-cAMP reversed the CB1R pattern distribution (Fig. 1).

Finally, all the analyzed sperm samples displayed three fluorescence patterns, that resulted independent of the culture conditions adopted and never exceeded 10% of the sperm population (Fig. 1).

### CB1R binding

Boar sperm cells show CB1R binding according to a saturable process, which is fully displaced by SR1, the selective CB1R antagonist [22]. Capacitated sperm cells show a ∼40% decreased CB1R binding with respect to control (Table 1), in keeping with a previous report [22]. Interestingly, we found that no treatment affected the binding of the cannabinoid receptor agonist [^3^H]CP55.940 in HBT-bic incubated spermatozoa, whereas exposure to BSA at the end of the same treatments increased the CB1R activity up to 40% (Table 1). These data confirm that cholesterol content alters ligand recognition by CB1R, and hence its binding properties in capacitated sperm much alike in nerve cells [35].

**Table 1:** CB1R binding activity in boar sperm cells capacitated under different conditions.

	*CB1R Binding Activity (fmol/mg proteins)*	
	−BSA	+BSA
	*Ejaculated spermatozoa*	
	553±50	550±50
	*In vitro incubated spermatozoa (HBT-bic)*	
Ctr	300±29^§^	420±33*
Met-AEA	322±30^§^	435±30*
SR1	355±36^§^	479±42*
8Br-cAMP	343±33^§^	463±46*
Met-AEA+SR1	307±31^§^	429±39*
Met-AEA+8Br-cAMP	358±36^§^	465±40*
SR1+8Br-cAMP	342±32^§^	478±41*

The values express the means ± SD of three independent experiments. The different superscripts indicate values significantly different (p<0.05) within each column.

### MC540 labelling of sperm properties

**Figure 2:**
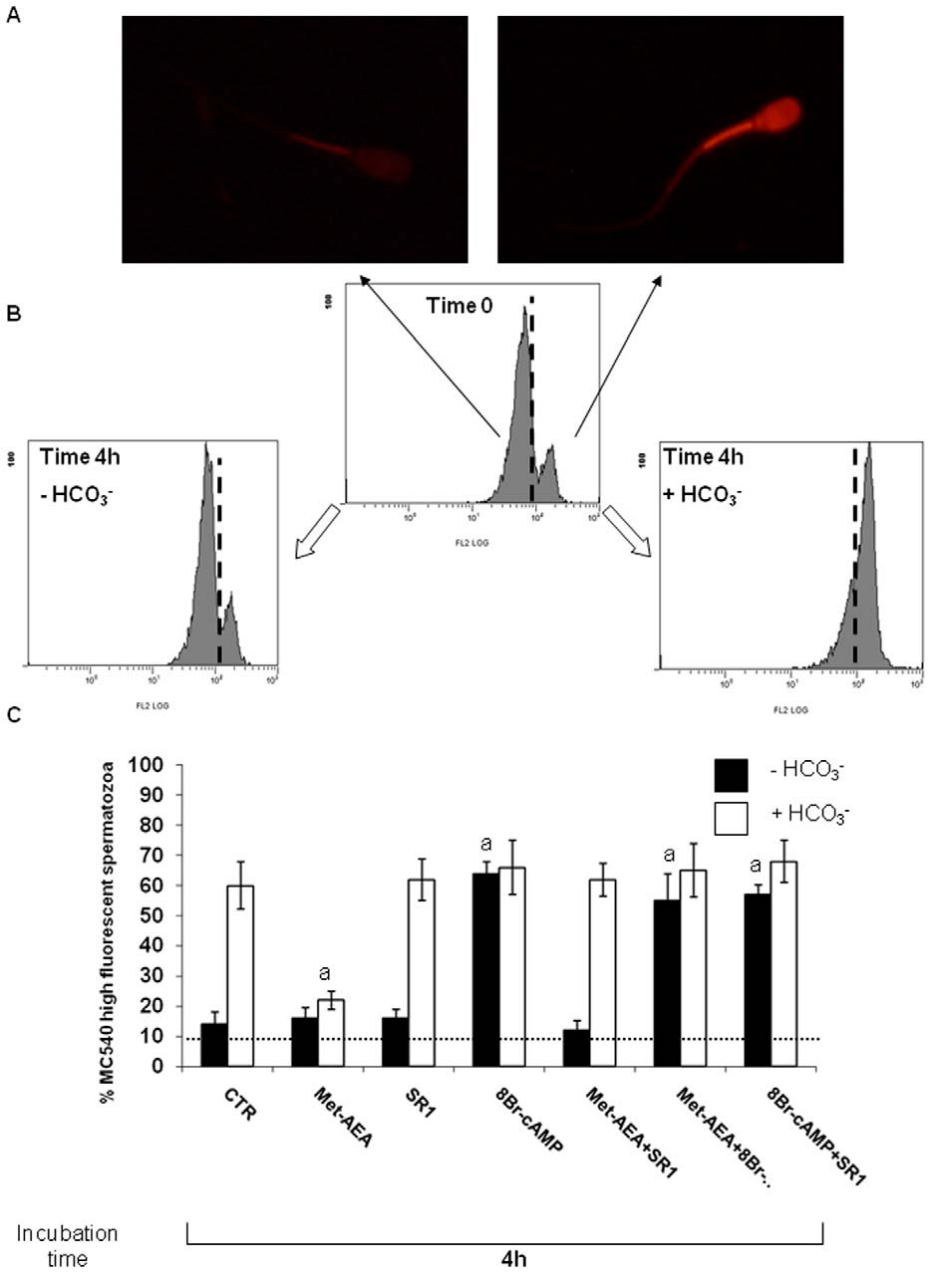
MC540 staining of spermatozoa incubated under capacitating conditions or upon different treatments. MC540 fluorescence increases with the increase of membrane scrambling. Panel A) Picture showing a MC540 negative spermatozoon (on the left) and a MC540 positive spermatozoon (on the right). Panel B) flow cytometry graphics showing the different population, low and high fluorescence emission (divided by black dot line) found in relation to different cultural conditions. Panel C) Effect of different treatments on the percentage of MC540 positive spermatozoa, at the beginning (Ejaculated sptz) and at the end of culture (In vitro incubated sptz). ^a^ = p<0.01 vs. other 4 h samples, ANOVA test.

MC540 staining revealed two different populations in ejaculated spermatozoa: one at low (∼80% of total cells), and one at high (∼20%) fluorescence (Fig. 2A and B).

The incidence of MC540 fluorescent patterns did not change in sperm cells incubated in HBT (Fig. 2B), while it significantly changed in the presence of either 8Br-cAMP or bicarbonate (HBT-bic). In fact, under both conditions the high fluorescence population became prevalent (∼60%: Fig. 2) at the end of culture.

The shift from low to high MC540 fluorescence pattern was totally prevented in Met-AEA treated sperm cells (Fig. 2C), where the incidence of highly fluorescent cells never exceeded ∼20%.

The inhibitory effect of Met-AEA was overcome by the addition of SR141716 (SR1) and of 8Br-cAMP (Fig. 2C). Instead, neither compound alone affected MC540 fluorescence properties of HBT-bic incubated sperm cells.

### Sperm cholesterol distribution

**Figure 3:**
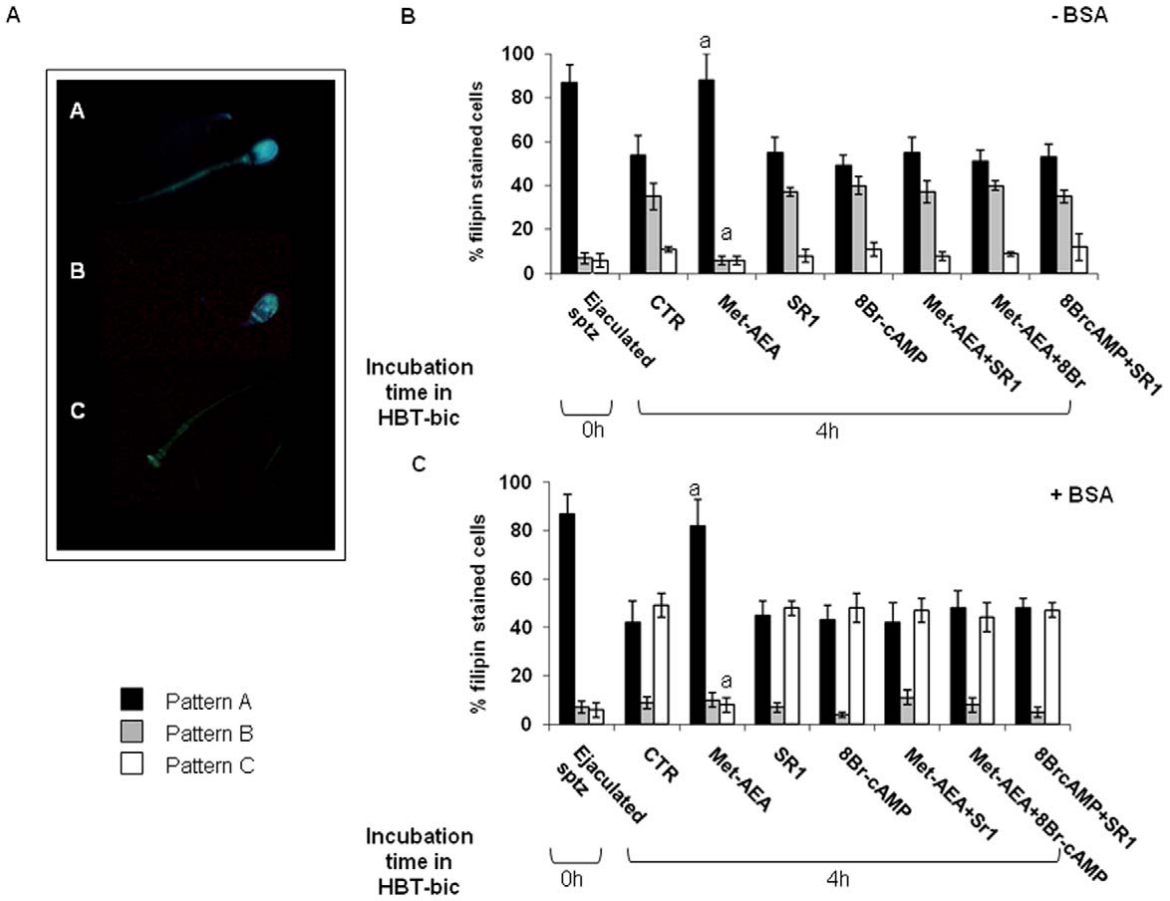
Different filipin staining patterns and their changes during the incubation under different treatments. Filipin makes complexes with cholesterol, thus allowing to visualize its distribution over the sperm membrane. Panel A) Different patterns of CB1R filipin staining found in boar spermatozoa. Panel B) Effect of different treatments on the percentage of different filipin patterns, at the beginning (Ejaculated sptz) and at the end of culture (In vitro incubated sptz), in absence of BSA. Panel C) Same as in panel B, in presence of BSA. ^a^ = p<0.01 vs. other 4 h samples, ANOVA test.

Ejaculated spermatozoa (data not shown) and HBT incubated cells (Fig. 3A) stained with filipin showed the following three different patterns:

- pattern A, filipin uniformly distributed over sperm head (79% and 82%, respectively),
- pattern B, fluorescent dye localized on the acrosomal region (7% and 9%, respectively),
- pattern C, absence of filipin over sperm head (6% and 4%, respectively).

The incidence of filipin fluorescence patterns changed when spermatozoa were incubated for 4 h in HBT-bic: pattern A dropped to ∼50%, pattern B increased ( >30%), and pattern C remained at an incidence of ∼5–10% (Fig. 3B). Similar sperm filipin labelling properties were recorded in spermatozoa incubated in HBT-bic supplemented with SR1, or in HBT and HBT-bic supplemented with 8Br-cAMP.

In the presence of Met-AEA the proportion of spermatozoa displaying pattern A, B and C remained similar to that recorded in ejaculated or HBT-incubated spermatozoa (∼80%, 5% and ∼5%, respectively; Fig. 3B). On the contrary, the filipin fluorescence pattern A and B in Met-AEA treated spermatozoa significantly changed, when incubations were performed in the presence of SR1 or of 8Br-cAMP (Fig. 3B).

In order to check whether the above described culture conditions were able to affect cholesterol location/extraction, sperm samples were briefly exposed to cholesterol acceptors such as BSA or MCD, 30 min before filipin staining (Fig. 3C).

Ejaculated (time 0) or HBT-incubated spermatozoa did not change their filipin labelling properties, when exposed to BSA (Fig. 3C). On the contrary, filipin staining was affected by BSA exposure when spermatozoa were incubated in HBT-bic media. Under the latter conditions, serum proteins induced a drop in the incidence of filipin pattern B (∼10%), paralleled by an increase of pattern C (>45%). BSA-induced cholesterol redistribution was completely absent in spermatozoa incubated in HBT-bic supplemented with Met-AEA, as demonstrated by the persistent high incidence of spermatozoa displaying filipin pattern A (<80%).

The addition of SR1 or 8Br-cAMP removed the effect of Met-AEA; in fact, a sharp increase in the percentage of sperm cells displaying pattern C (>45% and 50%, respectively; Fig. 3C) was recorded after BSA exposure.

Finally, the percentage of pattern C dramatically increased in all sperm samples exposed to MCD, independently of the incubation time or of the culture conditions adopted.

### Cholesterol content in culture media and sperm membrane-enriched fractions

The level of cholesterol was determined in incubation media and membrane-enriched fractions (MEF) after exposure of sperm to BSA or MCD.

As shown in Table 2, cholesterol levels in culture media were not affected by the conditions adopted. However, cholesterol content increased by ∼30–50% in all media obtained from spermatozoa incubated in HBT-bic exposed to BSA, except those collected from Met-AEA-treated cells. In fact, the levels of cholesterol recorded in the media obtained from spermatozoa incubated in HBT-bic supplemented with Met-AEA were similar to those collected from ejaculated spermatozoa exposed to BSA.

**Table 2:** Cholesterol content detected in HBT-bic media collected from spermatozoa incubated under different conditions.

	*Cholesterol content (µM)*	
	−BSA	+BSA
	*Ejaculated spermatozoa*	
	2.04±0.35	2.12±0.89
	*In vitro incubated spermatozoa (HBT-bic)*	
**Ctr**	2.08±0.40	2.96±0.33^a^
**Met-AEA**	2.12±0.38	2.07±0.40
**SR1**	2.00±0.44	2.99±0.22^a^
**8Br-cAMP**	1.99±0.23	3.02±0.26^a^
**Met-AEA+SR1**	2.01±0.25	2.98±0.32^a^
**Met-AEA+8Br-cAMP**	2.10±0.32	2.94±0.30^a^
**SR1+8Br-cAMP**	1.98±0.34	3.08±0.36

Values are expressed as means ± SD of three independent experiments, and the different superscripts indicate values significantly different within a column ( p<0.01).

The levels of cholesterol dramatically increased after sperm exposure to MCD, becoming 5.5 fold higher than those detected in media collected from incubated spermatozoa, independently of the culture conditions adopted.

**Figure 4:**
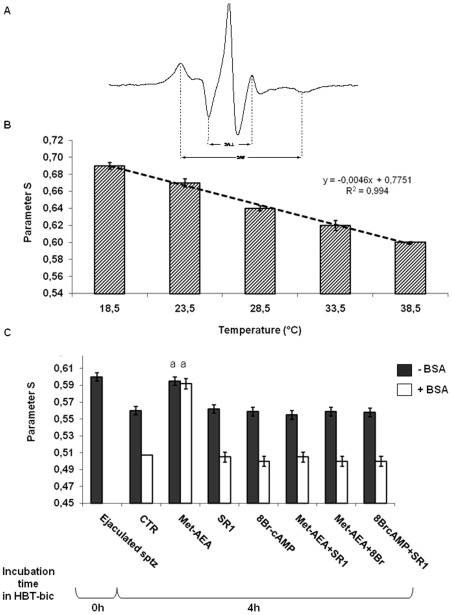
Assessment of parameter s during the incubation under different treatments. Panel A) Typical ESR graph. Panel B) Effect of different temperatures on parameter S. Panel C) Effect of different treatments on parameter S, at the beginning (Ejaculated sptz) and at the end of culture (In vitro incubated sptz), in the absence or in the presence of BSA. ^a^ = p<0.01 vs.other 4 h samples, ANOVA test. ^b^ = p<0.05 vs. other 4 h samples, ANOVA test.

The ratio between cholesterol and phospholipids (C/P) detected in MEF isolated from spermatozoa incubated under different culture conditions are summarized in Fig. 4. The C/P was higher in MEF isolated from ejaculated spermatozoa or sperm cells incubated in HBT (data not shown), or HTB-bic supplemented with Met-AEA (Fig. 4), while lower values of C/P were recorded in any other culture condition adopted.

A further decrease in C/P was recorded when HBT-bic incubated spermatozoa were exposed to BSA, independently of the culture conditions adopted. However, this drop in C/P values was significantly less evident in Met-AEA treated sperm cells. Finally, the C/P ratio dramatically dropped in all MEF exposed to MCD (data not shown).

### Membrane fluidity

**Figure 5:**
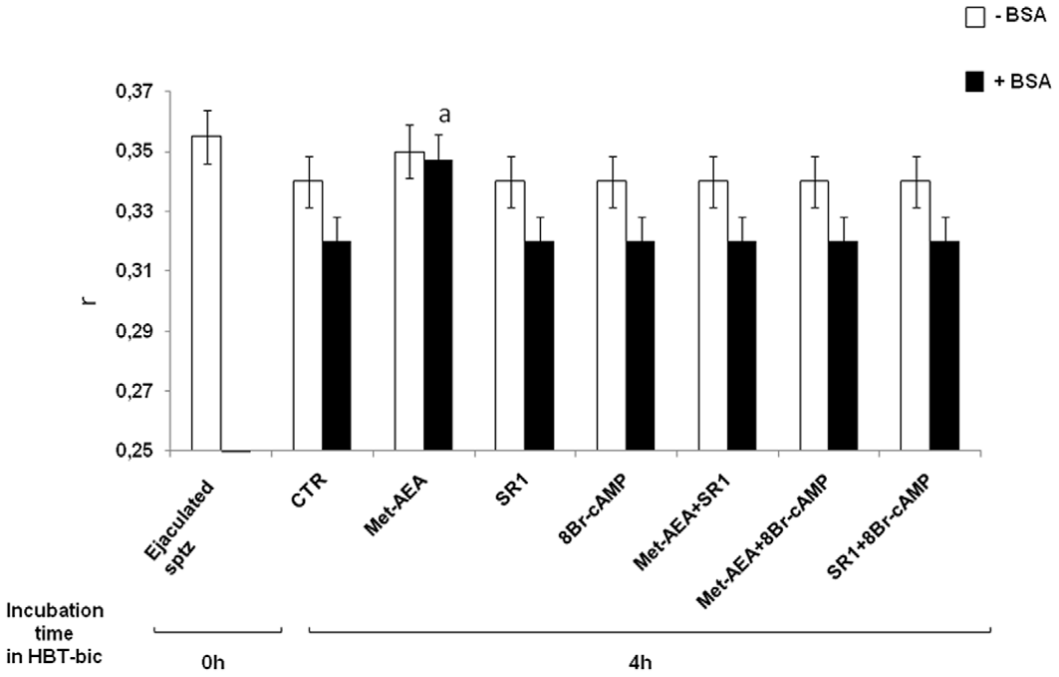
Assessment of membrane anisotropy (r) during the incubation under different treatments. Effect of different treatments on membrane anisotropy (r), at the beginning (Ejaculated sptz) and at the end of culture (In vitro incubated sptz), in the absence or in the presence of HCO_3_^−^ or BSA. ^a^ = p<0.01 vs. other 4 h samples, ANOVA test.

Since EPR spectrometry is not routinely applied to the study of sperm membrane fluidity, first of all, the variance of S parameter was analyzed by exposing boar spermatozoa to decreasing temperatures. As summarized in Fig. 5B, a linear correlation existed between S parameter and temperature, that could be expressed as S = −0.0046°C+0.7751 (R^2^ = 0.994, p<0.001). Then, EPR analysis was performed on sperm samples incubated under the different culture conditions, as well as before and after cholesterol extraction by BSA or MCD.

Ejaculated and HBT-incubated spermatozoa showed an S parameter ranging from 0.601 to 0.604, independently of the time of incubation and of the treatment with BSA (Fig. 5C).

By contrast, S parameter was lower in spermatozoa incubated in HBT-bic (0.562±0.005: p<0.01 vs. HBT incubated sperm cells), and further decreased after exposure to BSA (0.507±0.006: Fig. 5C) if Met-AEA was not present (0.595±0.005: p<0.01 vs. HBT-bic incubated spermatozoa).

The influence of Met-AEA was mediated by CB1R activation; indeed, SR1 or 8Br-cAM significantly reduced the S parameter down to values of 0.562±0.005 and 0.559±0.006, respectively (Fig. 5C). Finally, a clear decrease in S parameter was always recorded in spermatozoa exposed to MCD, down to values of 0.490–0.470 (Fig. 5C).

### Membrane anisotropy

**Figure 6:**
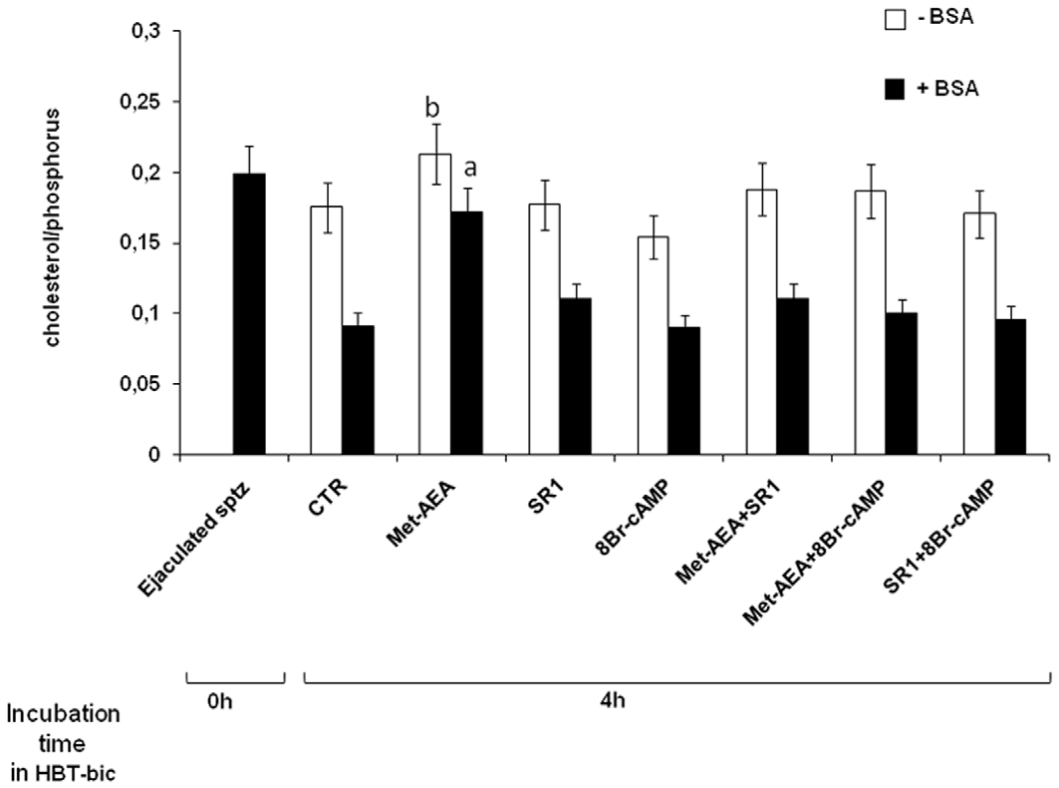
Effect of different treatments on membrane cholesterol/phosphorus ratio. Effect of different treatments on membrane cholesterol/phosphorus ratio, at the beginning(Ejaculated sptz) and at the end of culture (In vitro incubated sptz), in the absence or in the presence of HCO_3_^−^ or BSA. ^a^ = p<0.01 vs. other 4 h samples, ANOVA test.

The role of CB1R modulation on membrane anisotropy was evaluated in MEF loaded with the DPH (1,6-diphenyl-1,2,5-hexatriene) fluorescent probe, and the results are summarized in Fig. 6.

The *r* parameter was unaffected by BSA exposure of MEF of ejaculated and HBT-cultured spermatozoa (data not shown). The addition of bic promoted a slight decrease in *r* parameter in all sperm samples analyzed. BSA addition further decrease *r* parameter of MEF isolated from all groups of HBT-bic incubated sperm cells, except for those treated with Met-AEA.

As expected, MCD exposure promoted a dramatic drop of *r* parameter, independently of the culture conditions adopted.

### In Vitro Fertilization assay

The role exerted by CB1R activation on the sperm fertilizing ability was evaluated by an In Vitro Fertilization (IVF) assay. As shown in Table 3, the addition of Met-AEA to the capacitation medium caused a reduction in the number of fertilized oocytes to approximately one third (Ctr 90% vs. Met-AEA 33%, p<0.01), and an overt drop in the % of polyspermic zigotes (Ctr 60% vs. Met-AEA 40%, p<0.01) and in the number of sperm per oocyte (Ctr 4.7 vs. Met-AEA 2.7, p<0.01).

**Table 3:** Effect of Met-AEA on In Vitro Fertilization.

	Fertilization rate (%)	Polyspermic zigotes (%)	Number of sperm per oocyte
**Ctr**	90.3±4.9	60.3±3.5	4.7±0.6
**Met-AEA**	33.0±5.7^a^	40.8±5.5^a^	2.3±0.6^a^

Values are expressed as means ± SD of three independent experiments, and the different superscripts indicate values significantly different within a column ( p<0.01).

## Discussion

The spermatozoa of eutherian organisms can fertilize only after a complex series of functional and morphological modifications called capacitation. This process involves several mechanisms, most of which relate to remodelling of the physico-chemical asset of membranes [10], [11], [13]. Altogether these events, even if only partially known, seem to fulfil two important goals:

to organize the membrane antigenic mosaic, in order to specifically localize the signalling transduction machinery involved in the process of sperm-oocyte recognition, such as TSC4 [36] and OBF13 [37], T and S [38], head 37 kDa protein and tail 40 kDa [39], PH20, HSP70 [40] or TRPV1 [41];
to increase the fusogenic ability of sperm plasma membranes (PM and OAM), in order to allow exocytosis of the acrosome enzymatic content after ZP-binding [10], [11].

In this study, the effect of CB1R activation on this crucial process of lipid sperm membrane reorganization was investigated, first by assessing how the CB1R distribution and activity change during the process of *in vitro* capacitation and then by describing the role exerted by the receptor on the physico-chemical proprieties of sperm membranes.

The immunocytochemistry results showed that the incubation of spermatozoa under capacitating conditions caused a clear CB1R translocation from the post-acrosomal area (pattern A) to the equatorial region (pattern B). The evolution of the different fluorescent patterns and the results obtained with the functional test of sperm exposure to ZP strongly indicated that capacitated spermatozoa displayed pattern B. Moreover, the functional correlation between cAMP-dependent signalling pathways and CB1R translocation to the equatorial district highlighted the inhibitory role of the receptor on the process of sperm capacitation. In fact spermatozoa maintained their uncapacitated status until CB1R activity was high and the receptor was localized in the post acrosomal region. Both these conditions were associated either with the presence of high levels of AEA when the receptor antagonist is absent or with the activity of other inhibitory molecules able to maintain low the intracellular cAMP levels. Under physiological conditions, the post-acrosomal distribution of CB1R is preserved until spermatozoa are exposed to high levels of extracellular AEA, as it occurs in seminal plasma (ejaculated spermatozoa) and in the uterine district [28], [29]. Under these conditions, high levels of AEA may contribute to prevent a premature capacitation, by activating a CB1R-mediated reduction of intracellular cAMP. As the spermatozoa progress along the female genital tract, they are progressively exposed to lower concentration of AEA and higher of bicarbonate (a cAMP agonist), thus the brake exerted by CB1R could be removed. At the same time, the receptor becomes sequestrated in the equatorial region of the sperm (pattern B), where it could be associated with reduced activity. In addition, an autocrine/paracrine inhibitory feedback exists between intracellular cAMP levels and CB1R localization. In fact, cAMP concentration may control the transition from pattern A to pattern B and the AEA-mediated activation of CB1R modulates the cAMP levels, as demonstrated by the addition of SR1 or Met-AEA. On the other hand, the binding activity of CB1R was not affected by addition of Met-AEA, SR1, 8Br-cAMP or their combinations (Table 1), suggesting that capacitation modulates CB1R-dependent signalling essentially by controlling its intracellular localization.

We further ascertained whether CB1R activation could mediate membrane phospholipids/cholesterol remodelling. In a first set of experiments, membrane disorder (measured by MC540 staining) and cholesterol lateral distribution (indicated by filipin pattern B) were induced by the addition of bic. Under *in vitro* conditions the bicarbonate modified the physico-chemical properties of sperm membranes, increasing their fluidity either in alive spermatozoa (by ESR) or isolated MEF (by membrane anisotropy). The functional effect of bic on membrane fluidity was, at least in part, cholesterol independent, as indicated by the C/P ratios. These results were in agreement with Gadella and Harrison [18], who demonstrated that bic induces an apoptosis-unrelated exposure of phosphatidylserine and phosphatidylethanolamine in alive acrosome-intact spermatozoa. The reduction in C/P ratio may be stimulated by a protein kinase A-dependent intracellular pathway [17], thus causing the membrane disorder required for the completion of capacitation. In the present study, for the first time it was demonstrated that bic-dependent sperm activation can be completely prevented by Met-AEA, controlling plasma membrane phospholipid architecture in a CB1R-dependent manner. Incidentally, the effect of Met-AEA on lipid membrane distribution is in agreement with the hypothesis that cholesterol extraction is the endpoint of the control of membrane phospholipid disorder [10], [11]. In fact, BSA treatment did not induce any cholesterol extraction in sperm cells incubated in bic-Met-AEA, as indicated by the low cholesterol levels in the culture media, and did not influence membrane fluidity measured by ESR or by membrane anisotropy. Taken together, these data suggest that the complex biochemical machinery involved in the capacitation process is strictly controlled by endocannabinoids through CB1R activation, to such an extent that the CB1R antagonist SR141716 completely inhibited the effect of Met-AEA.

Accumulated evidence in different experimental paradigms has strengthened the concept that the cascade of events leading to sperm capacitation are spatio-temporally controlled through the activity of integrated biochemical mechanisms, as well as through the modulator influence of stimulating/inhibiting molecules. In this context, different and complementary techniques could help to unravel the complex events underlying the capacitation process. Here, ESR and membrane anisotropy techniques were used to collect informations on sperm membrane fluidity. It should be noted that both methods used probes (5-DSA and DPH, respectively) that bind membrane lipids in an unpredictable manner. Therefore it could be hypothesized that the binding properties of both the probes may be strictly dependent of sperm membrane composition and, as a consequence, of sperm functional state. For this reason, S or r parameters were compared and used to obtain a mean value of membrane fluidity, that may be the expression of the different membrane districts and of different sperm cell subpopulations. For this reason EPR and membrane anisotropy were integrated with filipin staining, a reliable qualitative approach yielding information on the incidence of different sperm subpopulations before and after cholesterol extraction. On the basis of these results obtained with different techniques, the reduction of S and *r* parameters following cholesterol extraction induced by MCD could be easily interpreted. Filipin measurements indicated, in fact, that this cyclic oligosaccharide was able to extract cholesterol from sperm membranes independently of the capacitation status and, therefore, of the preliminary disorder in phospholipid transmembrane distribution. On the contrary, BSA exposure was influenced by the priming effect of bic on phospholipid organization. Its ability to extract cholesterol, as indicated by combining P and r parameters, was inhibited in Met-AEA treated cells, when a lower incidence of pattern C of filipin fluorescence was recorded. In addition, the effect of cholesterol extraction from sperm membrane seems to confirm what observed in cellular models where cholesterol influence on membrane fluidity was correlated to the surrounding molecules [42]. Cholesterol extraction decreases membrane fluidity, when it is close to unsaturated fatty acid; instead, if saturated fatty acids are more abundant, cholesterol acts by promoting an increase in membrane fluidity and, as a consequence, by lowering membrane order (decrease in S parameter). The boar spermatozoa membrane contains relatively high amounts of unsaturated phospholipids [43] and, for this reason, cholesterol extraction induced by BSA or MCD reduced both S and *r* parameters.

**Figure 7:**
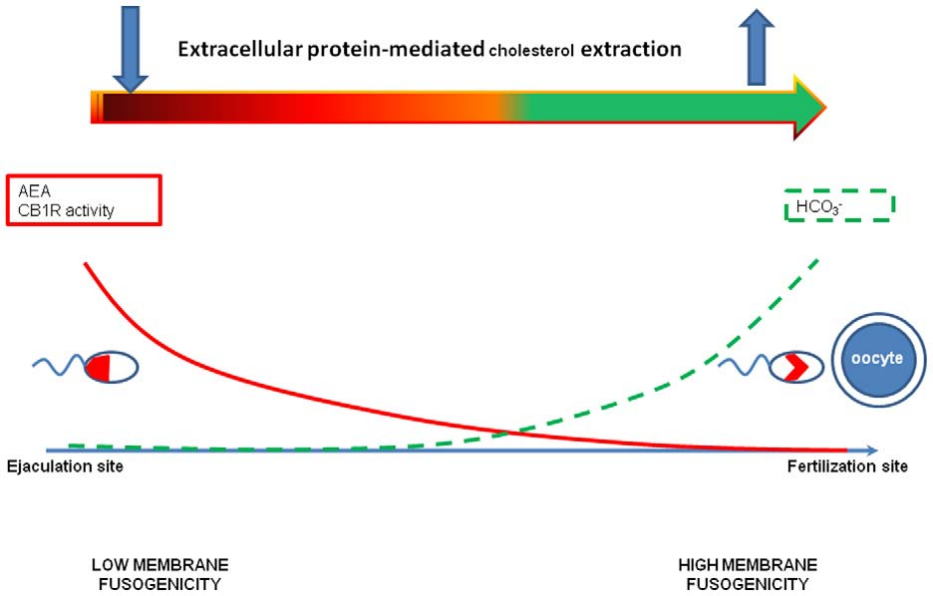
Effect of anandamide and bicarbonate gradients on sperm membrane physico-chemical properties. AEA concentration and CB1R binding activity decrease (red line) while the bicarbonate concentration increases (green dot line). In parallel, the localization of CB1R changes (from Pattern A to Pattern B) and the extracellular protein-mediated cholesterol extraction increases. As a consequence the membranes became more fusogenic.

Taken together, converging evidence supports the unprecedented concept that AEA can inhibit capacitation by preventing an increase in membrane fluidity, that is recognized as a key parameter involved in the acquisition of the fertilizing ability of spermatozoa. In fact, during capacitation the plasma membrane (PM) and the outer acrosome membrane (OAM) become less stable and gradually acquire the ability to fuse with each other (fusogenicity). If fusogenicity is not completely reached, the spermatozoa are unable to extrude the acrosome content in the presence of the oocyte's ZP, and thus fertilization does not take place. Instead, if the membrane fusogenicity increases, the AR could happen prematurely before the encounter between sperm and oocyte. Thus, the dialogue between sperm inhibiting and activating molecules may represent an extremely sensible strategy to guarantee the success of reproduction. In line with this, under physiological *in vivo* conditions mammalian spermatozoa after the ejaculation are progressively exposed to decreasing levels of AEA [28] and increasing levels of bic [15], thus passing from an inhibiting to a permissive condition during the transit from the uterus to the oviduct. This condition, in fact, progressively attenuates the brake exerted by the activation of CB1R, thus increasing the intracellular levels of cAMP and sequestering the inhibitory receptor in the equatorial region. The high levels of bic become able to activate sperm cells only within the oviduct, where the inhibitory effect of CB1R on cAMP intracellular concentration is lost (see Fig. 7). The observation that CB1R stimulation by Met-AEA was able to markedly decrease the fertilizing ability of boar sperm tested in IVF assays further corroborates this hypothesis.

In conclusion, the present research has improved our understanding of the role of the endocannabinoid system in the complex events leading to sperm-oocyte recognition. In the female genital tract, AEA can be a crucial element able to modulate sperm membrane response to bic gradient, and to control spatio-temporally the activation of spermatozoa. Apart from the possible physiological role of AEA in controlling sperm functionality, it is tempting to suggest that the data reported here may represent a novel platform of information on which new diagnostic and therapeutic strategies may be developed to treat male infertility and sperm management and storage.

## Materials and Methods

### Sperm preparation

The present animal work has been conducted according to relevant national and international guidelines that exempts this type of study from review. In particular boar semen was collected by the gloved-hand technique from three different males of proven fertility housed in the University farm and was prepared as previously described [22], [41]. The incubation was performed in Tyrode's media (HBT: 120 mM NaCl, 21.7 mM lactate, 20 mM Hepes, 5 mM glucose, 3.1 mM KCl, 2.0 mM CaCl_2_, 1.0 mM pyruvate, 0.4 mM MgSO_4_, 0.3 mM NaH_2_PO_4_ and 100 µg/ml kanamycin; 300 mOsm/kg, pH 7.4) with o without bicarbonate, in order to stimulate or prevent sperm activation. The media were supplemented with PVA and PVP (0.5%, respectively), in order to substitute serum proteins that could adsorb AEA and hence interfere with its biological activity. Incubation was then carried out at 1×10^8^ spermatozoa/ml (final concentration) for 4 h at 38.5°C in 5% CO_2_ humidified atmosphere (Heraeus, Hera Cell).

In order to modulate CB1R binding activity during incubation, the following treatments were performed:

addition of the non-hydrolysable analogue Met-AEA 1 mM, to mimic high extracellular levels of AEA;
inhibition of CB1R with the specific antagonist SR1 (100 mM) [22];
addition of the permeable analogue 8Br-cAMP (1 mM), in order to mimic high intercellular levels of cAMP [22].

At the end of the incubation, only samples showing a mean viability of ∼70% were used for further analysis. Sperm viability was assessed by using Hoechst 33258, as reported [41].

In order to evaluate the effect of the different culture conditions on cholesterol content and membrane fluidity/anisotropy, BSA (0.3%) or MCD(1 mM) were added for a short time (30 min) at the end of the incubation. Then, aliquots of control spermatozoa were exposed for 30 min to the solubilised ZP (sZP), in order to induce the acrosome reaction in capacitated spermatozoa.

### Immunocytochemical localization of CB1R

Immunocytochemistry was performed according to a previously reported protocol [22], that allowed to analyze CB1R distribution on sperm cells during the culture period.

In brief, sperm samples were washed, fixed and exposed to rabbit anti-CB1R polyclonal antibodies (1∶400; Cayman Chemicals, Ann Arbor, MI, USA) for 1 h at 38°C, and then to the secondary goat anti-rabbit CY3 conjugates (1∶400;Sigma Chemical Co., St. Louis, MO, USA) for 1 h at RT. After several washing steps, sperm samples were analysed with a fluorescence microscope (Nikon Eclipse E 600). For each treatment, at least 200 spermatozoa were recorded on three separate slides.

### Assay of CB1R binding

Cannabinoid receptor binding was analysed by rapid filtration assays, using a 96-wells plate and the synthetic cannabinoid [^3^H]CP55.940 (400 pM). In all experiments, unspecific binding was determined in the presence of the cold agonist (1 µM CP55.940) and was further corroborated by displacement through 0.1 µM SR1, a selective CB1R antagonist, as reported [22].

### M540 sperm staining

The merocyanine 540 (MC540) staining was adopted to assess the effect of CB1R-related pathways on sperm phospholipid membrane distribution [15], [17]. The MC540 florescence depends on the physical arrangement of the membrane (increasing with the increase in lipid disorder), and partially on the membrane potential, thus giving information on the lipid scrambling process [17]. Briefly, aliquots of alive sperm were stained with 2.7 µM MC540, and were assessed by flow cytometry (Beckman Coulter, Coulter Epics XL). A total of 10.000 sperm were analyzed in triplicate for each sperm treatment, using forward- and side- scatter profiles. Data obtained from flow cytometry were acquired and analyzed by EXPO 32 ADC software.

### Detection of sperm cholesterol distribution by filipin staining

Filipin staining was used with the aim of evaluating if CB1R activation could affect membrane cholesterol distribution and extraction. Filipin is a highly fluorescent probe that can aggregate choleterol into complexes visible under UV light. Sperm aliquots were fixed (4% glutaraldehyde in PBS) for 30 min, and then were exposed to 25 µM filipin according to Flesch et al. [17]. For each treatment at least 200 cells/slide were recorded in triplicate and analyzed under a fluorescence microscope (Nikon Eclipse E 600).

### Biochemical detection of cholesterol and phosphorus content of membrane-enriched fractions (MEF)

The “membrane-enriched fractions” (MEF) were prepared according to [44] with minor modifications. In brief, sperm samples were washed by centrifugation at 3600 *g* for 15 min in PBS, in order to resuspend the resulting pellet in a hypotonic buffer (2 mM Tris, pH 7.2, 12 mM NaCl) supplemented with the protease inhibitor cocktail (aprotinin, chymostatin, leupeptin and antipapain, 1 mM PMSF). The sperm suspension maintained on ice was, then, sonicated 6 times for 15 sec with 1 min intervals. Then, after a low-speed spin centrifugation (1600 *g* for 15 min), the resulting pellet was homogenized and the MEF were obtained as previously described [45]. A quantitative detection of sperm cholesterol content was performed on MEF [46], [47] by HPTLC, following anisaldehyde staining [48]. Spot identification and quantification was accomplished by comparison with authentic cholesterol standard, according to Palestini et al. [46]. In addition aliquots of MEF were used for phospholipids phosphorus determination by the Bartlett procedure [49].

### Membrane fluidity of living cells

The effect of different treatments on sperm membrane fluidity was estimated by means of electron paramagnetic resonance (ESR).

The ESR technique consists of embedding a nitroxide free-radical, 5-doxilstearic acid (5-DSA), into the membrane of interest, and then studying the free-radical order parameter, S. In fact, the nitroxide ESR spectrum line width is controlled by rotational and lateral diffusion of the spin probes. Broader line width results from the slower tumbling rate of the spin probe with a longer relaxation time. The S parameter gives a measure of the degree of structural order in the membrane, and is defined as the ratio of the spectral anisotropy in membranes obtained in a rigidly oriented system. For a rapid spin-label motion of about only one axis S = 1, and for a rapid isotropic motion S = 0. Parameter S can be calculated from the spectrum by means of the expression:
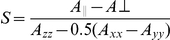
where 2A// and 2A⊥ are the separations between the outer and inner extrema in the experimental spectra, respectively (figure 5A), and A_zz_ A_xx_ and A_yy_ are the components of the hyperfine splitting tensor along the nitroxide principal axis (A_zz_ =  33.5 G, A_xx_ =  6.4 G, A_yy_  =  5.9 G). S values were not corrected for polarity. Samples were prepared according to [50] at final concentrations of 1×10^7^ cells/200 ml, for spermatozoa, and 10 nM, for 5-DSA. After spin probe loading, the samples were washed and aspirated in a glass capillary.

### Membrane anisotropy of MEF

In parallel experiments, fluorescence anisotropy measurements were used to assess the effect of different treatments on sperm membrane fluidity. To this aim, the fluorescent probe 1,6-diphenyl-1,2,5-hexatriene (DPH) was used, as previously described [46]. The linearly fluorescent probe DPH is a long and rigid molecule of rougly the shape and size of a fully extended phospholipid acyl chain. This probe has excitation and emission dipoles thar are roughly collinear with long molecular axes. When DPH is mixed with lipid-containig systems, it is incorporated very efficiently into the hydrocarbon core of the lipid; then, its rotation over time diminuishes the vertical components (Iper) and enhances the horizontal components (Ipa) of the analyzed light in a way described by the time-dipendence of fluorescence anisotropy (*r*). Briefly DPH was added, at a final concentration of 2 µM, to a MEF suspension containing ∼200 nmol phosphorus/1.5 ml TBS. Then, the MEF were maintained under stirring condition for 45 min at 37°C in the dark, before starting the fluorescence polarization measurements. A polarization spectrofluorimeter (Cary Eclipse, Varian) with fixed excitation and emission polarization filters to measure fluorescence intensity parallel (Ipa) and perpendicular (Iper) to the polarization plane of the exciting light [46] was used. Excitation and emission wavelengths were 360 and 430 nm, respectively. Light scattering was corrected by using a blank containing the sample incubated without DPH.

The fluorescence anisotropy was calculated as *r*, defined as follows:
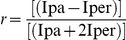



### In Vitro Fertilization assay

To study the effect of Met-AEA on sperm fertility, IVF assays were carried out. To this end, oocytes collection and maturation was performed as already described [51], [52] and the spermatozoa were incubated under capacitating conditions in the presence or absence of Met-AEA. At the end of capacitation, the spermatozoa were washed by centrifugation (800 g, 8 min) and the IVF was performed according to a previously validated protocol [51], [52]: in particular, to achieve the fertilization of at least part of the oocytes in each experimental group, and expecting an adverse effect of Met-AEA on the fertilizing ability of spermatozoa, an high concentration of cells (0.5×10^7^ motile spermatozoa/ml) was used. After 1 h of incubation in the presence of spermatozoa, the oocytes were gently removed from the Petri dish, transferred in fresh medium and maintained in culture for at least 8 h. Three independent determinations, each using 35–40 oocytes, where carried out. The effect of Met-AEA on the sperm fertilizing ability was quantified at the end of the incubation as the fertilization rate (% of penetrated oocytes), the incidence of polyspermy (% of polyspermic oocytes) and the mean number of penetrating spermatozoa per polyspermic oocyte, recorded according to Abeydeera [53] and Bernabò [51], [52]. To this aim, oocytes were mounted on a slide, were fixed in ethanol and acetic acid (3/1 v/v) and were assessed under a Nikon Eclipse E600 microscope (40×) after Lacmoid staining.

### Statistical analysis

Data reported in this paper are the mean (± S.D.) of 3 to 5 independent experiments, each performed in triplicate. To compare the effect exerted by different treatments, the data were checked for normal distribution by Shapiro-Wilks W test, and were compared by ANOVA test followed, if necessary, by *post-hoc* Tukey test (StatistiKL Version β). The differences were considered significant and highly significant for p values of <0.05 and <0.01, respectively. In the correlation test, a linear regression model or a Boltzmann sigmoidal model were used (Microcal Origin 6.0).
